# Order effects in stimulus discrimination challenge established models of comparative judgement: A meta-analytic review of the Type B effect

**DOI:** 10.3758/s13423-024-02479-3

**Published:** 2024-03-19

**Authors:** Ruben Ellinghaus, Karin M. Bausenhart, Dilara Koc, Rolf Ulrich, Roman Liepelt

**Affiliations:** 1 Department of General Psychology: Judgment, Decision Making, Action, Faculty of Psychology, University of Hagen (FernUniversität in Hagen), Hagen, Germany; 2 Department of Psychology, Cognition and Perception, University of Tuebingen, Tuebingen, Germany

**Keywords:** Stimulus discrimination, Psychophysics, Time order error, Comparative judgment, Type B effect, Stimulus history, Signal detection theory

## Abstract

This paper provides a comprehensive review of the Type B effect (TBE), a phenomenon reflected in the observation that discrimination sensitivity varies with the order of stimuli in comparative judgment tasks, such as the two-alternative forced-choice (2AFC) paradigm. Specifically, when the difference threshold is lower (higher) with the constant standard preceding rather than following the variable comparison, one speaks of a negative (positive) TBE. Importantly, prominent psychophysical difference models such as signal detection theory (Green & Swets, 1966) cannot easily account for the TBE, and are hence challenged by it. The present meta-analysis provides substantial evidence for the TBE across various stimulus attributes, suggesting that the TBE is a general feature of discrimination experiments when standard and comparison are presented successively. Thus, inconsistent with psychophysical difference models, subjective differences between stimuli are not merely a function of their physical differences but rather also depend on their temporal order. From the literature, we identify four classes of potential candidate theories explaining the origin of the TBE, namely (1) differential weighting of the stimulus magnitudes at the two positions (e.g., Hellström, *Psychological Research,* *39*, 345–388 1977), (2) internal reference formation (e.g., Dyjas, Bausenhart, & Ulrich, *Attention, Perception, & Psychophysics,* *74*, 1819–1841 2012), (3) Bayesian updating (e.g., de Jong, Akyürek, & van Rijn, *Psychonomic Bulletin and Review,* *28*, 1183–1190 2021), and (4) biased threshold estimation (García-Pérez & Alcalá-Quintana, *Attention, Perception & Psychophysics,* *72*, 1155–1178 2010). As these models, to some extent, make differential predictions about the direction of the TBE, investigating the respective boundary conditions of positive and negative TBEs might be a valuable perspective for diagnostic future research.

The more closely actual judgments are studied, the more evident does it become that they do not proceed according to the clean logical schemes which we are prone to devise for them in advance. Robert S. Woodworth, 1899, p. 818

Discriminating between stimuli that differ along one physical dimension (e.g., sound pressure, duration) constitutes one of the most fundamental cognitive abilities. It is therefore not surprising that psychophysicists have employed discrimination tasks vastly and in different areas of perception ever since the dawn of experimental psychology. Notably, to this day, the experimental procedures commonly used in this regard are often based on a classic procedure already employed by Gustav T. Fechner under the name *method of constant stimuli* (Fechner, 1860; Hegelmaier, 1852; Laming & Laming, 1992). The most prominent variant of this procedure might be the two-alternative forced-choice task (2AFC), originally developed by Hegelmaier (1852). In this task, a standard $s$ with constant magnitude and a comparison $c$ with magnitude varying from trial to trial are presented successively, and participants have to identify the larger stimulus at the end of each trial. The order of $s$ and $c$ varies randomly from trial to trial, yielding trials with order $\langle cs \rangle$ or order $\langle sc \rangle$. As explained below, one intriguing finding is that discrimination performance differs as a function of stimulus order. The goal of the present work is to provide a review of this temporal order effect, as well as a discussion of its theoretical implications.

## Measuring discrimination performance

**Fig. 1:**
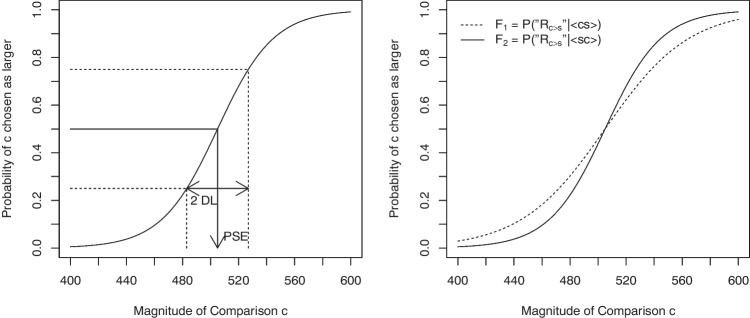
*Left plot*. Hypothetical logistic psychometric function of a 2AFC duration discrimination experiment with a standard $$s$$ of 500 msec. The location of the function defines the $$PSE$$, or point of subjective equality. Half the distance between the levels of c respectively corresponding to $$c_{25\%}$$ and $$c_{75\%}$$ (i.e., half the interquartile range) defines $$DL$$. Hence, $$DL$$ is a measure of sensitivity reflected in the steepness of the psychometric function. *Right plot*. Hypothetical order-dependent logistic psychometric functions $$ F_1 = P(R_{c>s} | \langle cs \rangle )$$ and $$ F_2 = P(R_{c>s} | \langle sc \rangle )$$ of a 2AFC duration discrimination experiment with a standard $$s$$ of 500 msec. Note that both functions have identical locations corresponding to an identical $$PSE$$ for both stimulus orders. However, $$ F_2$$ is steeper than $$ F_1$$, implying a larger $$DL$$ for $$\langle cs \rangle $$ trials than for $$\langle sc \rangle $$ trials and hence exhibiting a negative TBE

To measure discrimination sensitivity (i.e., the difference threshold) as obtained with this paradigm, one typically estimates the magnitude difference between $s$ and $c$, which enables identification of the larger stimulus with an accuracy level of 75% (Gescheider, 1997). This measure is conventionally defined as the difference limen ($DL$; or just noticeable difference $JND$). In order to estimate $DL$, a psychometric function is typically fitted to the data obtained from a discrimination experiment. This psychometric function plots the probability that $c$ is judged to be larger than $s$ on the *y*-axis as a function of the physical magnitude of $c$ on the *x*-axis. Psychometric functions typically increase from 0 to 1 with increasing values of $c$ (Fig. 1). Note that $DL$ is captured by the slope of the psychometric function. Specifically, half the distance between the levels of *c* corresponding to the 0.25 and 0.75 probabilities (i.e., half the interquartile range) defines $DL$. Thus, the steeper the psychometric function, the smaller the $DL$ and hence the higher the participant’s sensitivity.

Contrary to intuition, discrimination sensitivity as measured in this task is not merely a function of the physical difference between *s* and *c*, but also depends on their temporal order. To our knowledge, the first to notice this were Lillie J. Martin and Georg E. Müller when exploring weight discrimination (Martin & Müller, 1899). These pioneers of experimental psychology noted that participants more often judged correctly whether the second of two successively lifted weights was lighter or heavier than the first stimulus when $s$ preceded rather than followed $c$—implying higher discrimination performance in trials with stimulus order $\langle sc \rangle$ than $\langle cs \rangle$. In detail, they concluded that *“with the same effective difference, in those cases where the comparison weight was lifted second, more correct judgments (and also more correct judgments where the difference was more pronounced) were obtained compared to those cases where the comparison weight was lifted first“* (p. 25).^1^ However, although Robert S. Woodworth (Woodworth, 1899) praised the work of Lillie J. Martin and Georg E. Müller for demonstrating the actual complexity underlying seemingly trivial judgment procedures, this contribution was not widely received within the scientific community.

### Theoretical relevance of temporal order effects

It was about a hundred years later that psychophysicists not only rediscovered the effects of temporal order on discrimination sensitivity empirically but also realized their theoretical importance. For example, Nachmias (2006, p. 2462) noted the slope of the psychometric function to be steeper for $\langle sc \rangle$ than for $\langle cs \rangle$ trials. This observation of him reflects an instance of the *Type B effect* (TBE, Ulrich & Vorberg, 2009), and is illustrated in Fig. 1.^2^

Importantly, one speaks of a *negative* TBE if $DL$ is larger for $\langle cs \rangle$-trials than for $\langle sc \rangle$-trials, while a *positive* TBE refers to the opposite result pattern, i.e., smaller $DL$ for $\langle cs \rangle$-trials than for $\langle sc \rangle$-trials. Mainly negative TBEs have been reported in the literature (Bausenhart, Dyjas, & Ulrich, 2015; Dyjas et al., 2012; Ellinghaus, Ulrich, & Bausenhart, 2018; Rammsayer & Ulrich, 2012; Stott, 1935; Ulrich, 2010; Woodrow, 1935). Positive TBEs have mostly been reported for stimuli of short duration presented with very short interstimulus intervals (Hellström & Rammsayer, 2004, 2015; Hellström, 2000). Although the TBE has been mainly investigated for the case of duration discrimination (Ellinghaus, Gick, Ulrich, & Bausenhart, 2019; Hellström & Rammsayer, 2015; Woodrow, 1935), some studies have reported a TBE for physical dimensions outside the temporal domain (Ellinghaus et al., 2018; Ross & Gregory, 1964).^3^

The resurgence of interest in temporal order effects may be partially attributed to the formal psychophysical models that explicitly specify stimulus discrimination mechanisms. Many of these models, as for example signal detection theory (Green & Swets, 1966; Macmillan & Creelman, 2005; Wickens, 2002) and other prominent psychophysical models (Luce & Galanter, 1963; Yeshurun, Carrasco, & Maloney, 2008), are so-called *difference models* of stimulus discrimination. These models are rooted in the pioneering work of Thurstone (1927a, 1927b), who postulated that humans base their comparative judgment on the difference of the internal stimulus representations ${\textbf{D}}=\textbf{X}_1-\textbf{X}_2$, whereby $\textbf{X}_1$ and $\textbf{X}_2$ represent the internal magnitudes of the first and second stimulus in a trial. Crucially, these traditional difference models of the 2AFC task imply that the perceived magnitude difference ${\textbf{D}}$ on any trial does not depend on the stimulus order of *s* and *c* but merely on their magnitudes. Consequently, as shown by Dyjas et al. (2012), difference models cannot easily account for the TBE.

### Type B effect: Potential explanations

Various explanations have been proposed to account for the TBE. These might be subsumed under three separate theoretical frameworks. First, based on a suggestion by Durlach and Braida (1969), Nachmias (2006) reasoned that *“judgments would be based on comparing the second stimulus presented on the trial with some conglomerate of the virtual standard*^4^*and the first stimulus of the trial“*(p. 2462). Elaborating on this idea, Lapid, Ulrich, and Rammsayer (2008) and Dyjas et al. (2012) formalized a cognitive mechanism according to which such internal reference *I*, akin to Nachmias’s virtual standard, is established and updated on every trial. According to this internal reference model (IRM), participants complete their task in a current trial *n* by comparing the internal representation $\textbf{X}_{2,n}$ of the second stimulus in this trial against the current internal reference ${\textbf{I}}_n$, which is a conglomerate of previous and current stimulus instances and updates continuously from trial to trial. As the subjective difference ${\textbf{D}}_n$ on the present trial is the result of this comparison process, it can be stated as

1 $$\begin{aligned} {\textbf{D}}_n = {\textbf{I}}_n - \textbf{X}_{2,n}. \end{aligned}$$

Specifically, the internal reference ${\textbf{I}}_n = g \cdot {\textbf{I}}_{n-1} +(1-g) \cdot \textbf{X}_{1,n}$ on trial *n* is a weighted sum of the first stimulus’ internal representation $\textbf{X}_{1,n}$ on the current trial *n* and the internal reference ${\textbf{I}}_{n-1}$ from the previous trial $n-1$, with constant weight *g*, $0 \le g <1$. If ${\textbf{D}}_n >0$, then participants judge the first stimulus to be larger than the second stimulus, whereas when ${\textbf{D}}_n<0$, they judge the second stimulus to be larger than the first stimulus. Dyjas et al. (2012) demonstrated that this mechanism implies a better discrimination performance when $s$ precedes rather than follows $c$, and thus predicts negative TBE. Several related ideas have been proposed within the Bayesian framework (de Jong et al., 2021; Glasauer & Shi, 2021; Jazayeri & Shadlen, 2010; Schumacher & Voss, 2023; Shi, Church, & Meck, 2013; Wiener, Thompson, & Coslett, 2014), which are closely related to IRM (cf. de Jong et al., 2021). For completeness, it should be noted here that IRM and these related models have not only proven useful as potential accounts for the TBE, but may also explain some related temporal context effects such as central tendencies in judgment (e.g., Bausenhart, Dyjas, & Ulrich, 2014; Hollingworth, 1910; Vierordt, 1868) and assimilatory sequence effects (e.g., Cicchini, Arrighi, Cecchetti, Giusti, & Burr, 2012; Dyjas et al., 2012; Fischer & Whitney, 2014; Fritsche, Mostert, & de Lange, 2017) - for an integrative review see Sadibolova and Terhune (2022).

A second framework wherein which effects of stimulus order can be accounted for is the sensation weighting model (SWM; Hellström, 1977; Hellström, 1985; Hellström, 2000; Hellström & Rammsayer, 2004; Hellström & Rammsayer, 2015). As SWM is conceptually based on the adaptation level theory (Helson, 1947, 1964; Michels & Helson, 1954), it shares with IRM the premise that judgments rely on both past and present stimulus information. In contrast to IRM, SWM assumes that the magnitudes of the internal representations for the first and second stimuli are differently weighted when the stimuli are compared. Depending on the relative weights of the first and second stimulus, discrimination performance is higher or lower for the $\langle sc \rangle$ compared to $\langle cs \rangle$ (also see Bausenhart et al., 2015). Therefore, SWM can also account for a positive TBE (i.e., larger $DL$ for $\langle sc \rangle$ than for $\langle cs \rangle$ trials). As noted above, such positive TBEs have been reported by Hellström and his colleagues for duration discrimination experiments (e.g., Hellström & Rammsayer, 2004; Hellström, Patching, & Rammsayer, 2020).

Third, it has been argued that the TBE might not reflect any specific cognitive mechanism at all, but rather constitutes a methodological artifact which results from researcher decisions in estimating the difference threshold. For example, García-Pérez and Alcalá-Quintana (2010) argued that the higher difference thresholds in $\langle cs \rangle$ as compared to $\langle sc \rangle$ trials as observed by Lapid et al. (2008) are due to an supposedly erroneous procedure of $DL$ estimation. In detail, these authors on p. 1160 claim that “the wrong choice of a psychometric function to fit to 2AFC data, as well as the lack of a free lapsing-rate parameter, spuriously inflated DLs estimated by Lapid et al. (2008)“. While Ulrich (2010) doubted that the method employed by Lapid et al. (2008) is actually flawed, according to this interpretation of García-Pérez and Alcalá-Quintana (2010), a TBE should be evident (as an artifact) only in studies which employ a particular and supposedly incorrect procedure of $DL$ estimation.

In summary, the TBE has diagnostic value for evaluating the validity of psychophysical models. Specifically, as outlined above, TBEs are at variance with the predictions of traditional difference models but consistent with extensions of these models, such as IRM or SWM. Because these extensions generally apply to any stimulus discrimination task, the TBE was hypothesized to also systematically emerge outside the temporal domain. Coherently, the present meta-analysis includes both temporal and non-temporal studies in a random-effects model (Borenstein, Hedges, Higgins, & Rothstein, 2009).

## Method

### Sample of studies

Several papers addressing the effect of stimulus order on discrimination sensitivity for the 2AFC task have been published throughout the decades. The starting point for selecting studies was an overview provided by Dyjas et al. (2012). For the present analysis, we supplemented this preliminary compilation by adding more recent and earlier studies that had not been incorporated in this initial overview. Since authors have used various terms to refer to the TBE, keyword-based literature research would not yield meaningful results. Therefore, we attempted to identify all relevant publications using a snowball approach, and searched for additional relevant studies based on the references and citations of studies on the TBE. In essence, we searched for all 2AFC experiments that reported separate $DL$s for $\langle cs \rangle$ and $\langle sc \rangle$ trials, and contained sufficient information to compute the standardized mean difference (SMD, see below). The obtained studies were subdivided into temporal (i.e., duration) and non-temporal (e.g., brightness, loudness) discrimination experiments. Regarding temporal discrimination, this resulted in the following studies: Bausenhart et al. (2015), Bruno, Ayhan, and Johnston (2012), Dyjas et al. (2012), Dyjas et al. (2014), Dyjas and Ulrich (2014), Ellinghaus et al. (2019), Ellinghaus, Giel, Ulrich, and Bausenhart (2021), Gao, Miller, Rudd, Webster, and Jiang (2021), Gordon (1967), Grondin and McAuley (2009), Harrison, Binetti, Mareschal, and Johnston (2017), Hellström and Rammsayer (2004), Hellström and Rammsayer (2015), Hellström et al. (2020), Lapid et al. (2008), Marchman (1969), Rammsayer and Wittkowski (1990), Van Allen, Benton, and Gordon (1966), Thönes, Von Castell, Iflinger, and Oberfeld (2018), Ulrich (2010). Regarding experiments outside the temporal domain, the following studies were obtained: Ellinghaus et al. (2018), Ellinghaus et al. (2021), Lapid, Ulrich, and Rammsayer (2009), Nachmias (2006), Ross and Gregory (1964), von Castell, Hecht, and Oberfeld (2017).

**Fig. 2:**
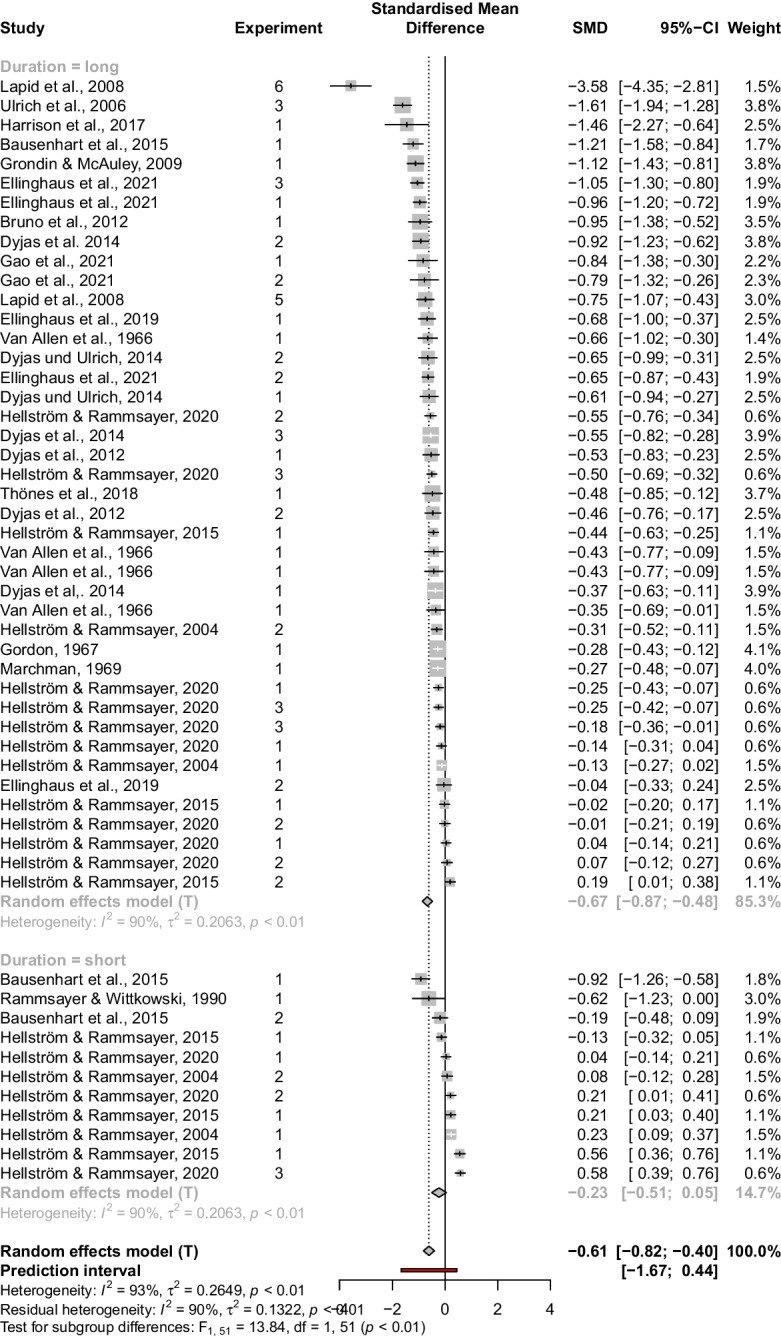
Results of temporal studies

### Coded factors and hypotheses

Two separate random-effects models were run. The first model included only the temporal studies and duration was used as a factor, with experiments employing an $s$ shorter or longer than 500 msecclassified as short or long, respectively. Theoretical considerations drove the inclusion of this factor. Namely, studies on duration discrimination suggest that the processing of very short durations relies on different mechanisms than the processing of longer durations. For example, Michon (1985) assumed that temporal processing of intervals longer than 500 ms is cognitively mediated, whereas shorter intervals are perceptual in nature. This distinct timing hypothesis received both neuroscientific (Lewis & Miall, 2003b, a) and behavioral support (Rammsayer & Lima, 1991; Rammsayer & Ulrich, 2011)—however, see Rammsayer and Ulrich (2005) for contradicting results. Consistent with the distinct timing hypothesis and earlier studies (cf. Bausenhart et al., 2015; Hellström & Rammsayer, 2004), we conjectured that the TBE will be negative at a relatively long interval length but become more positive at a relatively short interval length. Second, an additional random-effects model included only the non-temporal studies. Here, we expected the SMD to be significantly smaller than 0, reflecting substantial evidence for the negative TBE.

**Fig. 3:**
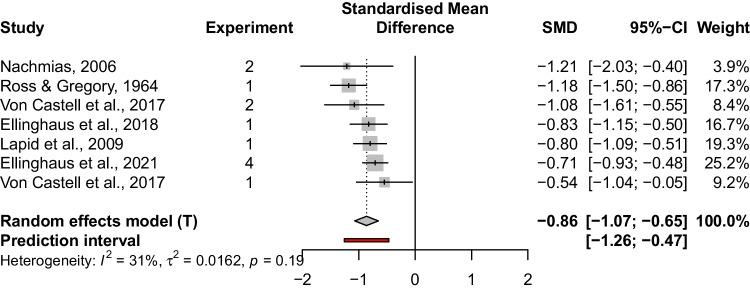
Results of non-temporal studies

### Effect size analysis

In a first step, the standardized mean difference (SMD, computational details are given by Borenstein et al., 2009) was computed for each documented TBE, defined as $\mathbf {D = DL_{<sc>}- DL_{<cs>}}$. For within-subjects designs, the employed expression was $\widehat{SMD} = \frac{M_D}{S_{within}}$, with $S_{within} = \frac{S_D}{\sqrt{2(1-r)}}$.^5^ For between-subjects designs, the employed expression was $\widehat{SMD} = t \cdot \sqrt{\frac{N_1 + N_2}{N_1 \cdot N_2}}$. In a second step, these estimated effect sizes of both the temporal and non-temporal stimuli were submitted to a random-effects model using the function metagen() of the R package meta (Schwarzer et al., 2007). For both analyses, the corresponding model accounted for the hierarchically nested data structure, that is, in each model each observed TBE was nested within a certain (sub)sample of a certain experiment, which was nested within a certain study. For both models, SMD was estimated across all observations. In addition, for the temporal studies, a subgroup analysis with the factor interval length was carried out to get separate SMD estimates for short and long stimulus durations. Finally, the function forest.meta() was employed to design the forest plots.

## Results

An overview of the temporal studies is given in Fig. 2. In the corresponding model, the SMD was estimated to be -0.61 (95% CI [-0.82; -0.40]) when averaged across all observations, and this estimate was significantly different from zero, $t(52) = -5.83$, $p < .001$. In addition, the subgroup analysis revealed a significantly more negative SMD for long durations (-0.67, 95% CI [-0.87; -0.48]) as compared to short durations (-0.23, 95% CI [-0.51; 0.05]), $F(1,51) = 13.84$, $p < .01$. An overview of the non-temporal studies is given in Fig. 3. The corresponding SMD was estimated as -0.86 (95% CI [-1.07; -0.65]), and significantly larger than zero, $t(6) = -10.05$, $p < .001$.

## Discussion

The goal of the present article was to review the literature on the TBE, i.e., the finding that discrimination performance as indexed by $DL$ differs as a function of the temporal order of $s$ and $c$ in the 2AFC task. Importantly, since the TBE had been primarily investigated for the temporal domain (i.e., duration discrimination), it was unclear whether this effect is the signature of some general cognitive process related to comparative judgments or specific to duration discrimination. Investigating this generality is theoretically important since the TBE poses a problem for traditional difference models (Green & Swets, 1966; Macmillan & Creelman, 2005; Wickens, 2002). Since these models are formulated rather generally, their predictions should hold across various stimulus attributes. Therefore, the present meta-analysis aimed to assess the TBE’s generality by including and comparing effect sizes of both temporal and non-temporal studies in a random-effects model (Borenstein et al., 2009).

The main results of this analysis can be summarized as follows: First, the meta-analytic regression model indicated substantial evidence for the TBE. Hence, in contrast to the predictions of traditional difference models, the subjective difference between two stimuli compared is not merely a function of their physical difference but also depends on their temporal order. Given that the analysis procedures of the 2AFC studies published throughout the decades and incorporated in the present analysis are very heterogeneous, it seems unlikely that the TBE merely reflects the consequences of a particular erroneous $DL$ estimation procedure, as claimed by García-Pérez and Alcalá-Quintana (2010). Rather, the TBE appears to constitute a real phenomenon with a mechanistic origin. As such, it challenges established models of stimulus discrimination, and can thus be considered a benchmark effect to elaborate these models. Notably, while most TBEs reported in the literature stem from the temporal domain, based on our analysis, it seems plausible that the TBE is an inherent feature of discrimination experiments where standard and comparison are presented successively, as the present analysis provides substantial evidence for the TBE in various non-temporal tasks such as line length or brightness discrimination. Based on our analysis, it seems plausible that the Type B effect is an inherent feature of each 2AFC experiment. In fact, the absolute value of the estimated SMD for the non-temporal domain seems numerically even slightly larger than for the temporal domain. A potential reason why this phenomenon may have been underrepresented in the literature so far is the common practice of averaging data from the two stimulus presentation orders in 2AFC experiments (see Ulrich & Vorberg, 2009). This practice is not advisable, however, as these authors have shown that neglecting order effects can distort $DL$ estimates. Computational tools for estimating discrimination performance that avoid such pitfalls are given by Bausenhart, Dyjas, Vorberg, and Ulrich (2012).

Second, while most TBEs correspond to the direction as predicted by IRM and related Bayesian updating models, that is, higher $DL$ for $\langle cs \rangle$ than for $\langle sc \rangle$ trials (hence reflecting a negative TBE), positive TBEs (higher $DL$ for $\langle sc \rangle$ than for $\langle cs \rangle$ trials) have also been documented (Hellström & Rammsayer, 2015). Notably, all of these documented positive TBEs stem from duration discrimination experiments, most of which employed very short standard durations (<100 ms). Coherently, when the factor interval length was employed as a moderator (short vs. long durations) in the meta-analytic regression model for the duration discrimination studies, the effect size of the TBE differed significantly between experiments respectively employing short versus long standard durations. This result is theoretically important, because positive TBEs are inconsistent with the predictions of IRM and related models (but see Bausenhart et al., 2015), yet can be accounted for by SWM. In detail, SWM can predict lower $DL$ for $\langle cs \rangle$ than for $\langle sc \rangle$ trials by postulating that the stimulus magnitude in the first stimulus position is given more weight than the magnitude in the second position. Nevertheless, it should be noted that the estimated mean SMD for the short duration temporal studies was still negative, and hence the present meta-analysis does not provide sufficient evidence to discard IRM in favor of SWM. It should also be noted that the number of experiments employing standard durations not longer than 100 ms is relatively small and only a few replication attempts outside the lab of Hellström and colleagues have been made, some of which were unsuccessful in replicating a positive TBE (Bausenhart et al., 2015; Rammsayer & Wittkowski, 1990).

In any case, even if the positive TBEs documented for short standard durations constitute a true effect, this does not generally falsify the mechanisms as proposed by IRM and related models in favor of SWM. Rather, it is possible that the scope of these models is not as general as previously hypothesized. For example, it is possible that the nature of integrating past and present stimulus information differs between relatively long and relatively short durations. In fact, it is a prominent idea in the psychology of time that different mechanisms operate in different time scales (Lewis & Miall, 2003b; Michon, 1985). Coherently, effects analogous to the TBE (different decision weights for the two stimulus positions) have been reported as increasingly positive (greater impact of the first stimulus than of the second) with brief durations and brief ISI (e.g., Hellström, 1979; Hellström, 2003).^6^ As the stimulus conditions employed in these experiments are rarely used by researchers, a biased view might arise when making inferences from the current literature. Clearly, investigating the exact boundary conditions under which positive and negative TBEs, respectively, occur will be crucial for future theory building research. Finally although not directly relevant to the TBE and its origin, it is worth mentioning that from the models considered here only SWM can genuinely account for the classic time order error. 7

In this article, we focused on the slope of the psychometric function, which is captured by the difference limen ($DL$, Luce & Galanter, 1963). It is theoretically possible that the order of $s$ and $c$ not only affects $DL$ but also higher moments of the psychometric function, such as the skewness of the function (cf. Ulrich, 1987). Unfortunately, we are not aware of any study that assessed potential order-effects for higher moments of the psychometric function. For future research, it might be valuable to also address higher moments of the psychometric function as a benchmark for evaluating models of stimulus discrimination.^8^

In conclusion, the present meta-analysis reveals that the TBE is a ubiquitous feature of discrimination tasks when a constant standard and a variable comparison are presented successively, as for example in the classic 2AFC task. This effect constitutes a challenge for classic difference models, such as SDT (e.g., Green & Swets, 1966) and other prominent psychophysical difference models (e.g., Yeshurun et al., 2008). Potential candidate mechanisms underlying the TBE are (1) differential weighting of the stimulus magnitudes at the two positions (e.g., Hellström, 1977), (2) internal reference formation (e.g., Dyjas et al., 2012), (3) Bayesian updating (e.g., de Jong et al., 2021), and (4) biased threshold estimation (García-Pérez & Alcalá-Quintana, 2010). In any case, future studies are needed to better understand under which conditions positive and negative TBEs, respectively, result, to more clearly delineate the underlying mechanisms of discrimination performance.

## Open Practices Statement

The data and analysis script of this meta-analysis will be made available upon publication of this article.

## Footnotes

1 “Die obigen Ergebnisse bedeuten also kurz gesagt, dass bei gleicher wirksamer Differenz in denjenigen Fällen, wo das Vergleichsgewicht zuzweit gehoben wurde, mehr richtige Urteile (und auch mehr richtige Urteile, wo der Unterschied ein deutlicher war) erhalten wurden als in denjenigen Fällen, wo das Vergleichsgewicht an erster Stelle gehoben wurde.“

2 Ulrich and Vorberg (2009) introduced the notion *Type B effect* to distinguish it from the classic time order error (Fechner, 1860; Hellström, 1985, for a comprehensive historical overview), which in their terminology is termed *Type A effect*.

3 It should be noted that the TBE emerges whether the order of $s$ and $c$ is randomized within the same block of trials or between blocks (see Dyjas et al., 2012).

4 He conceived the virtual standard to be a memory representation formed from similar events close in time and space.

5 Since the correlation *r* is normally not reported in the selected studies, it was approximated with 0.75 as reported for the experiments by Bausenhart et al. (2015). When the analyses reported here were repeated with an *r* of 0.5 or 0.3, no different conclusions were reached.

6 Based on SWM, the TBE is equivalent to different decision weights for the stimuli at the two positions. That is, the impact of the comparison stimulus on the response is proportional to its weight, and hence the $DL$ is inversely proportional to this weight (Hellström, 2000). However, as this measure does not adhere to our $DL$-based definition of the TBE, studies reporting this measure were not included in the meta-analytic regression model. Still, these experiments should be regarded as important evidence for order effects on discrimination performance (see also Oberfeld, 2015).

7 When experimenting on the discrimination of two successively lifted weights, Fechner observed that the first weight tended to be underestimated relative to the second weight – a phenomenon which came to be known as the negative time order error (Hellström, 1979, 1985; Stott, 1935; Woodrow, 1935; Guilford, 1954). Under specific circumstances, the first stimulus is overestimated relative to the second stimulus (i.e., a positive time order error, Fechner, 1860; Köhler, 1923; Woodrow, 1933). The origin of the time order effect has long been debated; while some theories propose a response bias (i.e., a preference for one of the two response alternatives) as its cause (Yeshurun et al., 2008; Dyjas et al., 2012), other theories assume a genuine perceptual origin of the effect (Hellström, 1977, 1985; Jamieson & Petrusic, 1975b, c, a; Killeen & Grondin, 2022; Köhler, 1923; Lauenstein, 1933). Independent of its exact underlying mechanism, the time order error becomes evident in a lateral shift of the psychometric function and hence a shift of the $PSE$ for the two stimulus orders $\langle sc \rangle$ and $\langle cs \rangle$.

8 This idea was inspired by an anonymous reviewer.

## References

[CR1] Bausenhart, K. M., Dyjas, O., & Ulrich, R. (2014). Temporal reproductions are influenced by an internal reference: Explaining the Vierordt effect. *Acta Psychologica,**147*, 60–67. 23896562 10.1016/j.actpsy.2013.06.011

[CR2] Bausenhart, K. M., Dyjas, O., & Ulrich, R. (2015). Effects of stimulus order on discrimination sensitivity for short and long durations. *Attention, Perception, & Psychophysics,**77*, 1033–1043. 10.3758/s13414-015-0875-8 25832187

[CR3] Bausenhart, K. M., Dyjas, O., Vorberg, D., & Ulrich, R. (2012). Estimating discrimination performance in two-alternative forced-choice tasks: Routines for MATLAB and R. *Behavior Research Methods,**44*, 1157–1174. 22773433 10.3758/s13428-012-0207-z

[CR4] Borenstein, M., Hedges, L. V., Higgins, J. P., & Rothstein, H. R. (2009). *Introduction to meta-analysis*. John Wiley & Sons.

[CR5] Bruno, A., Ayhan, I., & Johnston, A. (2012). Effects of temporal features and order on the apparent duration of a visual stimulus. *Frontiers in Psychology,**3*(90), 1–7. 10.3389/fpsyg.2012.00090 PMC3309524 22461778

[CR6] Cicchini, G. M., Arrighi, R., Cecchetti, L., Giusti, M., & Burr, D. C. (2012). Optimal encoding of interval timing in expert percussionists. *The Journal of Neuroscience,**32*, 1056–1060. 22262903 10.1523/JNEUROSCI.3411-11.2012 PMC6621155

[CR7] de Jong, J., Akyürek, E. G., & van Rijn, H. (2021). A common dynamic prior for time in duration discrimination. *Psychonomic Bulletin and Review,**28*, 1183–1190. 33661470 10.3758/s13423-021-01887-z PMC8367937

[CR8] Durlach, N. I., & Braida, L. D. (1969). Intensity perception. I. Preliminary theory of intensity resolution. *The Journal of the Acoustical Society of America,**46*, 372–383. 5804107 10.1121/1.1911699

[CR9] Dyjas, O., Bausenhart, K. M., & Ulrich, R. (2012). Trial-by-trial updating of an internal reference in discrimination tasks: Evidence from effects of stimulus order and trial sequence. *Attention, Perception, & Psychophysics,**74*, 1819–1841. 10.3758/s13414-012-0362-4 23055085

[CR10] Dyjas, O., Bausenhart, K. M., & Ulrich, R. (2014). Effects of stimulus order on duration discrimination sensitivity are under attentional control. *Journal of Experimental Psychology: Human Perception and Performance,**40*, 292–307. 23895391 10.1037/a0033611

[CR11] Dyjas, O., & Ulrich, R. (2014). Effects of stimulus order on discrimination processes in comparative and equality judgements: Data and models. *The Quarterly Journal of Experimental Psychology,**67*, 1121–1150. 24295428 10.1080/17470218.2013.847968

[CR12] Ellinghaus, R., Gick, M., Ulrich, R., & Bausenhart, K. M. (2019). Decay of internal reference information in duration discrimination: Intertrial interval modulates the Type B effect. *Quarterly Journal of Experimental Psychology,**72*, 1578–1586. 10.1177/1747021818808187 30282525

[CR13] Ellinghaus, R., Giel, S., Ulrich, R., & Bausenhart, K. M. (2021). Humans integrate duration information across sensory modalities: Evidence for an amodal internal reference of time. *Journal of Experimental Psychology: Learning, Memory, and Cognition,**47*, 1205.

[CR14] Ellinghaus, R., Ulrich, R., & Bausenhart, K. M. (2018). Effects of stimulus order on comparative judgments across stimulus attributes and sensory modalities. *Journal of Experimental Psychology: Human Perception and Performance,**44*, 7–12. 29309193 10.1037/xhp0000495

[CR15] Fechner, G. T. (1860). *Elemente der Psychophysik*. Leipzig, Germany: Breitkopf und Härtel.

[CR16] Fischer, J., & Whitney, D. (2014). Serial dependence in visual perception. *Nature Neuroscience,**17*, 738–743. 24686785 10.1038/nn.3689 PMC4012025

[CR17] Fritsche, M., Mostert, P., & de Lange, F. P. (2017). Opposite effects of recent history on perception and decision. *Current Biology,**27*, 590–595. 28162897 10.1016/j.cub.2017.01.006

[CR18] Gao, Y., Miller, K. N., Rudd, M. E., Webster, M. A., & Jiang, F. (2021). Duration comparisons for vision and touch are dependent on presentation order and temporal context. *Frontiers in integrative neuroscience,**15*, 664264. 34248513 10.3389/fnint.2021.664264 PMC8261066

[CR19] García-Pérez, M. A., & Alcalá-Quintana, R. (2010). Reminder and 2AFC tasks provide similar estimates of the difference limen: A re-analysis of the data from Lapid, Ulrich, & Rammsayer (2008) and a discussion of Ulrich & Vorberg (2009). *Attention, Perception & Psychophysics,**72*, 1155–1178. 10.3758/APP.72.4.1155 20436208

[CR20] Gescheider, G. A. (1997). *Psychophysics: The fundamentals (3rd)*. Mahwah, New Jersey: Lawrence Erlbaum Associates.

[CR21] Glasauer, S., & Shi, Z. (2021). The origin of Vierordt’s law: The experimental protocol matters. *PsyCh Journal,**10*, 732–741. 10.1002/pchj.464 34028202

[CR22] Gordon, M. C. (1967). Reception and retention factors in tone duration discriminations by brain-damaged and control patients. *Cortex,**3*, 233–249.

[CR23] Green, D. M., & Swets, J. A. (1966). *Signal detection theory and psychophysics* (Rev.). Los Altos, CA: Peninsula Publishing, reprinted Edition 1988.

[CR24] Grondin, S., & McAuley, J. D. (2009). Duration discrimination in crossmodal sequences. *Perception,**38*, 1542–1559. 19950485 10.1068/p6359

[CR25] Guilford, J. P. (1954). *Psychometric methods (2nd)*. New York: McGraw-Hill.

[CR26] Harrison, C., Binetti, N., Mareschal, I., & Johnston, A. (2017). Time-order errors in duration judgment are independent of spatial positioning. *Frontiers in Psychology,**8*, 340. 28337162 10.3389/fpsyg.2017.00340 PMC5343025

[CR27] Hegelmaier, F. (1852). Ueber das Gedächtniss für Linear-Anschauungen. *Archiv für physiologische Heilkunde,**11*, 844–853.

[CR28] Hellström, Å. (1977). Time errors are perceptual. *Psychological Research,**39*, 345–388.

[CR29] Hellström, Å. (1979). Time errors and differential sensation weighting. *Journal of Experimental Psychology: Human Perception and Performance,**5*, 460–477. 528952

[CR30] Hellström, Å. (1985). The time-order error and its relatives: Mirrors of cognitive processes in comparing. *Psychological Bulletin,**97*, 35–61.

[CR31] Hellström, Å. (2000). Sensation weighting in comparison and discrimination of heaviness. *Journal of Experimental Psychology: Human Perception and Performance,**26*, 6–17. 10696602 10.1037//0096-1523.26.1.6

[CR32] Hellström, Å. (2003). Comparison is not just subtraction: Effects of time- and space-order on subjective stimulus difference. *Perception & Psychophysics,**65*, 1161–1177. 14674641 10.3758/bf03194842

[CR33] Hellström, Å., Patching, G. R., & Rammsayer, T. H. (2020). Sensation weighting in duration discrimination: A univariate, multivariate, and varied-design study of presentation-order effects. *Attention, Perception, & Psychophysics,**82*, 3196–3220. 10.3758/s13414-020-01999-z PMC7381453 32342344

[CR34] Hellström, Å., & Rammsayer, T. H. (2004). Effects of time-order, interstimulus interval, and feedback in duration discrimination of noise bursts in the 50- and 1000-ms ranges. *Acta Psychologica,**116*, 1–20. 15111227 10.1016/j.actpsy.2003.11.003

[CR35] Hellström, Å., & Rammsayer, T. H. (2015). Time-order errors and standard-position effects in duration discrimination: An experimental study and an analysis by the sensation-weighting model. *Attention, Perception, & Psychophysics,**77*, 2409–2423. 10.3758/s13414-015-0946-x 26082151

[CR36] Helson, H. (1947). Adaptation-level as frame of reference for prediction of psychophysical data. *The American Journal of Psychology,**60*, 1–29. 20288861

[CR37] Helson, H. (1964). *Adaptation-level theory*. New York: Harper & Row.

[CR38] Hollingworth, H. (1910). The central tendency of judgment. *The Journal of Philosophy, Psychology and Scientific Methods,**7*, 461–469.

[CR39] Jamieson, D. G., & Petrusic, W. M. (1975a). Pairing effects and time-order errors in duration discrimination. *Perception & Psychophysics,**18*, 107–113.

[CR40] Jamieson, D. G., & Petrusic, W. M. (1975b). Presentation order effects in duration discrimination. *Perception & Psychophysics,**17*, 197–202.

[CR41] Jamieson, D. G., & Petrusic, W. M. (1975c). The dependence of time-order error direction on stimulus range. *Canadian Journal of Psychology/Revue Canadienne de Psychologie,**29*, 175. 10.1037/h0082023 1175095

[CR42] Jazayeri, M., & Shadlen, M. N. (2010). Temporal context calibrates interval timing. *Nature Neuroscience,**13*, 1020–1026. 20581842 10.1038/nn.2590 PMC2916084

[CR43] Killeen, P. R., & Grondin, S. (2022). A trace theory of time perception. *Psychological Review,**129*, 603. 10.1037/rev0000308 34553968

[CR44] Köhler, W. (1923). Zur Theorie des Sukzessivvergleichs und der Zeitfehler. *Psychological Research,**4*, 115–175.

[CR45] Laming, D., & Laming, J. (1992). F. Hegelmaier: On memory for the length of a line. *Psychological Research,**54*, 233–239. 1494608 10.1007/BF01358261

[CR46] Lapid, E., Ulrich, R., & Rammsayer, T. H. (2008). On estimating the difference limen in duration discrimination tasks: A comparison of the 2AFC and the reminder task. *Perception & Psychophysics,**70*, 291–305. 18372750 10.3758/pp.70.2.291

[CR47] Lapid, E., Ulrich, R., & Rammsayer, T. H. (2009). Comparisons of two variants of the method of constant stimuli for estimating difference thresholds. *Swiss Journal of Psychology,**68*, 189–192.

[CR48] Lauenstein, O. (1933). Ansatz zu einer physiologischen Theorie des Vergleichs und der Zeitfehler. *Psychologische Forschung,**17*, 130–177.

[CR49] Lewis, P. A., & Miall, R. C. (2003a). Distinct systems for automatic and cognitively controlled time measurement: Evidence from neuroimaging. *Current opinion in neurobiology,**13*, 250–255. 10.1016/s0959-4388(03)00036-9 12744981

[CR50] Lewis, P. A., & Miall, R. C. (2003b). Brain activation patterns during measurement of sub-and supra-second intervals. *Neuropsychologia,**41*, 1583–1592. 10.1016/s0028-3932(03)00118-0 12887983

[CR51] Luce, R. D., & Galanter, E. (1963). Discrimination. In R. D. Luce, R. R. Bush, & E. Galanter (Eds.), *Handbook of mathematical psychology* (Vol. 1, pp. 191–243). New York: John Wiley & Sons.

[CR52] Macmillan, N. A., & Creelman, C. D. (2005). *Detection theory: A user’s guide (2nd)*. Mahwah, New Jersey: Lawrence Erlbaum Associates.

[CR53] Marchman, J. N. (1969). Discrimination of brief temporal durations. *The Psychological Record,**19*, 83–92.

[CR54] Martin, L., & Müller, G. E. (1899). Zur Analyse der Unterschieds-empfindlichkeit [on the analysis of discriminal sensitivity]. Leipzig, Germany: Barth.

[CR55] Michels, W. C., & Helson, H. (1954). A quantitative theory of time-order effects. *The American Journal of Psychology,**67*, 327–334. 13158649

[CR56] Michon, J. A. (1985). The compleat time experiencer. In M. J.A. & J. J.L. (Eds.), *Time, mind, and behavior* (pp. 21–52). Berlin: Springer.

[CR57] Nachmias, J. (2006). The role of virtual standards in visual discrimination. *Vision Research,**46*, 2456–2464. 16530243 10.1016/j.visres.2006.01.029

[CR58] Oberfeld, D. (2015). Are temporal loudness weights under top-down control? effects of trial-by-trial feedback. *Acta Acustica United with Acustica,**101*, 1105–1115.

[CR59] Rammsayer, T. H., & Lima, S. D. (1991). Duration discrimination of filled and empty auditory intervals: Cognitive and perceptual factors. *Perception & Psychophysics,**50*, 565–574. 1780204 10.3758/bf03207541

[CR60] Rammsayer, T. H., & Ulrich, R. (2005). No evidence for qualitative differences in the processing of short and long temporal intervals. *Acta psychologica,**120*, 141–171. 15907778 10.1016/j.actpsy.2005.03.005

[CR61] Rammsayer, T. H., & Ulrich, R. (2011). Elaborative rehearsal of nontemporal information interferes with temporal processing of durations in the range of seconds but not milliseconds. *Acta Psychologica,**137*, 127–133. 21474111 10.1016/j.actpsy.2011.03.010

[CR62] Rammsayer, T. H., & Ulrich, R. (2012). The greater temporal acuity in the reminder task than in the 2AFC task is independent of standard duration and sensory modality. *Canadian Journal of Experimental Psychology,**66*, 26–31. 21910520 10.1037/a0025349

[CR63] Rammsayer, T. H., & Wittkowski, K. M. (1990). Zeitfehler und Positionseffekt des Standardreizes bei der Diskrimination kurzer Zeitdauern [Time-order error and position effect of the standard stimulus in the discrimination of short durations]. *Archiv für Psychologie,**142*, 81–89. 2092645

[CR64] Ross, H. E., & Gregory, R. L. (1964). Is the Weber fraction a function of physical or perceived input? *Quarterly Journal of Experimental Psychology,**16*, 116–122.

[CR65] Sadibolova, R., & Terhune, D. B. (2022). The temporal context in bayesian models of interval timing: Recent advances and future directions. *Behavioral Neuroscience*. 10.1037/bne0000513 PMC9552499 35737557

[CR66] Schumacher, L., & Voss, A. (2023). Duration discrimination: A diffusion decision modeling approach. *Attention, Perception, & Psychophysics,**85*, 560–577. 10.3758/s13414-022-02604-1 PMC9935725 36690915

[CR67] Schwarzer, G., et al. (2007). Meta: An R package for meta-analysis. *R news,**7*, 40–45.

[CR68] Shi, Z., Church, R. M., & Meck, W. H. (2013). Bayesian optimization of time perception. *Trends in Cognitive Sciences,**17*, 556–564. 24139486 10.1016/j.tics.2013.09.009

[CR69] Stott, L. H. (1935). Time-order errors in the discrimination of short tonal durations. *Journal of Experimental Psychology,**18*, 741–766.

[CR70] Thönes, S., Von Castell, C., Iflinger, J., & Oberfeld, D. (2018). Color and time perception: Evidence for temporal overestimation of blue stimuli. *Scientific Reports,**8*, 1–8. 29374198 10.1038/s41598-018-19892-z PMC5786107

[CR71] Thurstone, L. L. (1927a). A law of comparative judgment. *Psychological Review,**34*, 273–286.

[CR72] Thurstone, L. L. (1927b). Psychophysical analysis. *American Journal of Psychology,**38*, 368–389. 3322058

[CR73] Ulrich, R. (1987). Threshold models of temporal-order judgments evaluated by a ternary response task. *Perception & Psychophysics,**42*, 224–239. 3671048 10.3758/bf03203074

[CR74] Ulrich, R. (2010). DLs in reminder and 2AFC tasks: Data and models. *Attention, Perception, & Psychophysics,**72*, 1179–1198. 10.3758/APP.72.4.1179 20436209

[CR75] Ulrich, R., & Vorberg, D. (2009). Estimating the difference limen in 2AFC tasks: Pitfalls and improved estimators. *Attention, Perception & Psychophysics,**71*, 1219–1227. 10.3758/APP.71.6.1219 19633337

[CR76] Van Allen, M. W., Benton, A. L., & Gordon, M. C. (1966). Temporal discrimination in brain-damaged patients. *Neuropsychologia,**4*, 159–167.

[CR77] Vierordt, K. (1868). *Der Zeitsinn nach Versuchen*. Tübingen, Germany: Verlag der H. Laupp’schen Buchhandlung.

[CR78] von Castell, C., Hecht, H., & Oberfeld, D. (2017). Measuring perceived ceiling height in a visual comparison task. *Quarterly Journal of Experimental Psychology,**70*, 516–532. 10.1080/17470218.2015.1136658 26822335

[CR79] Wickens, T. D. (2002). *Elementary signal detection theory*. Oxford: Oxford University Press.

[CR80] Wiener, M., Thompson, J. C., & Coslett, H. B. (2014). Continuous carryover of temporal context dissociates response bias from perceptual influence for duration. *PloS one,**9*, e100803. 10.1371/journal.pone.0100803 PMC4071004 24963624

[CR81] Woodrow, H. (1933). Weight-discrimination with a varying standard. *The American Journal of Psychology,**45*, 391–416.

[CR82] Woodrow, H. (1935). The effect of practice upon time-order errors in the comparison of temporal intervals. *Psychological Review,**42*, 127–152.

[CR83] Woodworth, R. S. (1899). Zur Analyse der Unterschiedsempfindlichkeit. By Lillie J. Martin und GE Müller. Leipzig, JA Barth. 1899. pp. vii 233. M. 7.50. *Science,**10*, 818–819.

[CR84] Yeshurun, Y., Carrasco, M., & Maloney, L. T. (2008). Bias and sensitivity in two-interval forced choice procedures: Tests of the difference model. *Vision Research,**48*, 1837–1851. 10.1016/j.visres.2008.05.008 PMC5839130 18585750

